# Morphometric Analysis of Structural MRI Using Schizophrenia Meta-analytic Priors Distinguish Patients from Controls in Two Independent Samples and in a Sample of Individuals With High Polygenic Risk

**DOI:** 10.1093/schbul/sbab125

**Published:** 2021-10-18

**Authors:** Thomas M Lancaster, Stavros I Dimitriadis, Gavin Perry, Stan Zammit, Michael C O’Donovan, David E Linden

**Affiliations:** Department of Psychology, Bath University, Bath, UK; Cardiff University Brain Research Imaging Centre (CUBRIC), School of Psychology, Cardiff University, Cardiff, UK; Cardiff University Brain Research Imaging Centre (CUBRIC), School of Psychology, Cardiff University, Cardiff, UK; MRC Centre for Neuropsychiatric Genetics and Genomics, Institute of Psychological Medicine and Clinical Neurosciences, Cardiff School of Medicine, Cardiff University, Cardiff, UK; Cardiff University Brain Research Imaging Centre (CUBRIC), School of Psychology, Cardiff University, Cardiff, UK; MRC Centre for Neuropsychiatric Genetics and Genomics, Institute of Psychological Medicine and Clinical Neurosciences, Cardiff School of Medicine, Cardiff University, Cardiff, UK; Centre for Academic Mental Health, Population Health Sciences, Bristol Medical School, University of Bristol, Bristol, UK; MRC Centre for Neuropsychiatric Genetics and Genomics, Institute of Psychological Medicine and Clinical Neurosciences, Cardiff School of Medicine, Cardiff University, Cardiff, UK; Neuroscience and Mental Health Research Institute, Cardiff University, Cardiff, UK; Cardiff University Brain Research Imaging Centre (CUBRIC), School of Psychology, Cardiff University, Cardiff, UK; MRC Centre for Neuropsychiatric Genetics and Genomics, Institute of Psychological Medicine and Clinical Neurosciences, Cardiff School of Medicine, Cardiff University, Cardiff, UK; Centre for Academic Mental Health, Population Health Sciences, Bristol Medical School, University of Bristol, Bristol, UK; School of Mental Health and Neuroscience, Faculty of Health, Medicine and Life Sciences, Maastricht University, Maastricht, The Netherlands

**Keywords:** multivariate, MRI, normative modelling, schizophrenia, heterogeneity, polygenic

## Abstract

Schizophrenia (SCZ) is associated with structural brain changes, with considerable variation in the extent to which these cortical regions are influenced. We present a novel metric that summarises individual structural variation across the brain, while considering prior effect sizes, established via meta-analysis. We determine individual participant deviation from a within-sample-norm across structural MRI regions of interest (ROIs). For each participant, we weight the normalised deviation of each ROI by the effect size (Cohen’s *d*) of the difference between SCZ/control for the corresponding ROI from the SCZ Enhancing Neuroimaging Genomics through Meta-Analysis working group. We generate a morphometric risk score (MRS) representing the average of these weighted deviations. We investigate if SCZ-MRS is elevated in a SCZ case/control sample (*N*_CASE_ = 50; *N*_CONTROL_ = 125), a replication sample (*N*_CASE_ = 23; *N*_CONTROL_ = 20) and a sample of asymptomatic young adults with extreme SCZ polygenic risk (*N*_HIGH-SCZ-PRS_ = 95; *N*_LOW-SCZ-PRS_ = 94). SCZ cases had higher SCZ-MRS than healthy controls in both samples (Study 1: *β* = 0.62, *P* < 0.001; Study 2: *β* = 0.81, *P* = 0.018). The high liability SCZ-PRS group also had a higher SCZ-MRS (Study 3: *β* = 0.29, *P* = 0.044). Furthermore, the SCZ-MRS was uniquely associated with SCZ status, but not attention-deficit hyperactivity disorder (ADHD), whereas an ADHD-MRS was linked to ADHD status, but not SCZ. This approach provides a promising solution when considering individual heterogeneity in SCZ-related brain alterations by identifying individual’s patterns of structural brain-wide alterations.

## Introduction

Meta-analyses demonstrate that schizophrenia (SCZ) is associated with brain alterations detectable by structural magnetic resonance imaging (MRI). The Enhancing Neuroimaging Genomics through Meta-Analysis (ENIGMA) working group show that SCZ is associated with a wide range of regional MRI-derived brain alterations across an extensive cortical/subcortical landscape.^1–3^ As there is considerable overlap between structural indices of SCZ and control samples, several thousand SCZ-case/controls are needed to identify these individual ROI effects in independent samples which are limiting factors for both research studies and diagnostic applications. The comparison of any single regional brain metric may underestimate the extent of differences between patients and SCZ controls, due to the heterogeneity between patients, where alterations are not necessarily present in a uniform cortical pattern across all patients. Emerging evidence supports extensive regional heterogeneity for SCZ-related alterations in brain structure and symptomology.^4,5^ The structural alterations observed in meta-analysis of SCZ-patients may therefore describe a range of inter-individual variation, where specific anatomical loci are inconsistent across SCZ patients.^6^ Therefore novel, multivariate metrics capable of summarising brain alterations while considering sample and individual participant heterogeneity will help to capture inter and intra-participant variability across a population.

ENIGMA’s approach has helped to identify robust structural brain alteration in SCZ with similar success as the Psychiatric Genetics Consortium SCZ working group which identified effects of genetic variants associated with SCZ.^7,8^ Here, it is possible to combine the *en masse* effects of thousands of risk alleles with small effect into a single metric called a polygenic risk score (PRS),^8^ which can summarise the combined impact of all known/present risk loci for an individual. In contrast to single risk alleles which have negligible effects on liability (and offer limited power to distinguish between cases and controls), the SCZ-PRS captures a substantial fraction of liability (currently 7% on the liability scale, based on the median SCZ-PRS effect size from 40 target subgroups) in European populations,^9^ although significantly less in populations with ethnic disparity.^10,11^ Inspired by the PRS approach in genomics, we have developed a “morphometric risk score” (MRS). The MRS represents the combined, weighted combination of structural MRI alterations, where the weights are effects from the independent ENIGMA SCZ working group meta-analysis and individual brain regions are constrained by cytoarchitectural boundaries.^12,13^ Here, we assess an individual’s whole brain-based risk for SCZ based on each ROIs deviation from a wider sample norm, weighted by the proposed impact of established priors, such as ENIGMA-SCZ ROI effect sizes, as opposed to discrete metrics such as number of risk alleles used to estimate a SCZ-PRS. This approach builds upon metrics such as the regional-vulnerability index (RVI), which demonstrates that individuals with ROI deviations more similar to the effect sizes observed in SCZ case/control analysis are more likely to have a SCZ diagnosis.^14–17^ However, we aim to assess each ROI independently, rather than correlating all ROI/meta-analysis effect sizes, per individual. We aim to assess whether the combined influence of these weighted deviations as estimated by the MRS are associated with SCZ case status (versus controls). We further aim to determine if the MRS is also associated with genetic liability to schizophrenia, as assessed by SCZ-PRS in a healthy sample, as this would suggest that SCZ-related structural MRI alterations would have a causal role in SCZ aetiology. We thus aim to identify individuals with “schizophrenia-like brain alterations”, accounting for differences between individual SCZ patients/SCZ-PRS groups. We propose that using regional SCZ effect sizes derived from the ENIGMA-SCZ working group consensus will improve the power to detect SCZ-related brain alterations in independent samples, akin to how SCZ-PRS can distinguish between SCZ and controls in the absence of identifying individual genetic variant effects.^18^ While prior studies have used normative modelling approaches to distinguish SCZ case from controls,^6^ we anticipate the additional use of robust SCZ-priors effect size weights will help parse individual SCZ patient-specific heterogeneity, by accounting for individual profiles of structural alterations and provide disorder-specific sensitivity.

## Methods

### Participants

#### Study 1: Consortium for Neuropsychiatric Phenomics (CNP) cohort (Schizophrenia vs. healthy controls)

The CNP sample was used to compare structural MRI data from 50 patients with schizophrenia (SCZ, age 37.20 ± 9.16 years, 28 female/22 male) and 125 healthy controls (HCs, age 31.67 ± 8.81 years, 71 female/54 male). All participants provided written informed consent following procedures approved by the IRBs at UCLA and the Los Angeles County Department of Mental Health. The CNP sample was recruited from the greater Los Angeles area. Control subjects were excluded if they had a life-time diagnosis of an axis-I disorder, substance abuse or significant medical illness. Detailed sample and pre-processing descriptions are available for this public dataset^19,20^ available to download at: https://openneuro.org/datasets/ds000030/versions/1.0.0.

#### Study 2: Conte Center for the Neuroscience of Mental Disorders (CCNMD) cohort (Schizophrenia vs. healthy controls)

The CCMND sample was used to compare structural MRI data from 23 patients with schizophrenia (SCZ, age 24.25 ± 3.74 years, 6 female/17 male) and 20 healthy controls (HCs; age 20.66 ± 5.15 years, 8 female/12 male). All participants provided written informed consent for participation, reported in prior publications.^21^ Control subjects were excluded if they had a life-time diagnosis of an axis-I disorder, substance abuse or significant medical illness. Detailed sample and pre-processing descriptions are available for this public dataset^22^ available to download at: https://openneuro.org/datasets/ds000115/versions/00001.

#### Study 3: Recall-by-genotype (RBG) cohort (high SCZ-PRS vs. low SCZ-PRS).

The Avon Longitudinal Study of Parents and Children (ALSPAC) cohort characteristics and genotyping are described in supplementary materials. Construction of the SCZ-PRS follows the methods described by the International Schizophrenia Consortium,^7,8^ using results from the Psychiatric Genomics Consortium Wave 2 data release.^7^ Polygenic scores were calculated for each ALSPAC individual using the “score” command in PLINK (version 1.07).^23^ Individual SCZ-PRS were created by summing the number of risk alleles present for each SNP (0, 1, or 2) weighted by the logarithm of each SNP’s OR for SCZ from the PGC summary statistics. Our SCZ-PRS-based recall-by-genotype (RBG) was based upon a PRS generated from SNPs with a GWAS training-set *P* ≤ 0.05 threshold, chosen as it captures the maximum SCZ liability in the primary GWAS.^7^ From 8168 individuals with genotype data, we ascertained 189 (95 with high SCZ-PRS, 94 with low SCZ-PRS). Participants were invited/recruited to this sub-study if their SCZ-PRS was extremely high or low, compared to SCZ-PRS distribution across the wider ALSPAC cohort.^24,25^ Participants were recruited if their SCZ-PRS was at least 1 standard deviation above (high) or below (low) the ALSPAC SCZ-PRS mean. Further details about the RBG sample can be found in the sample description.^26^ Compared to the mean SCZ-PRS in the ALSPAC sample, (*N* = 8168, *Z*_SCZ-PRS_ = 0.00 ± 0.98) our SCZ-PRS groups had an average standard deviation of *Z*_SCZ-PRS_ = 1.41 ± 0.58 (high SCZ-PRS) and *Z*_SCZ-PRS_ = −1.71±0.46 (low SCZ-PRS). The SCZ-PRS groups were matched for sex (low SCZ-PRS: 48 female/46 male; high SCZ-PRS: 51 female/44 male).

### Neuroimaging acquisition and analysis

Structural T1 MRI data were acquired in three separate neuroimaging studies/samples, with scanning/acquisition parameters detailed in table 1. In alignment with SCZ-ENIGMA analysis strategies,^2,3^ we extracted subcortical volume (mm^3^), cortical thickness (mm) and surface area (mm^2^) from 75 regions of interest (34 bilateral cortical (× thickness and surface area) and 7 bilateral subcortical volumes) using Desikan–Killlany atlases for segmentation in FreeSurfer.^13,27^ We consider the independent influence of subcortical volume, cortical surface area and thickness due to their distinct phenotypic and genetic aetiology.^28–30^ Segmented subcortical and cortical regions were visually inspected and statistically evaluated for outliers following standardized ENIGMA protocols, where structural MRI segmentations that fall outside of 1.5 × interquartile (Q1–Q3) range are visually inspected (http://enigma.ini.usc.edu/protocols/imaging-protocols). All data were analysed independently, in a site-specific manner to minimize confounding from site-effects on MRI metrics.^31^

**Table 1.** T1-weighted structural MRI sequences/parameters across studies

Study	Sequence	Scanner	TR (s)	TE (ms)	Flip Angle	FOV (mm)	Voxel size (mm)	FreeSurfer version
1	MPRAGE	3T Siemens Trio	1.9	2.26	90°	256 × 256 × 250	1	6.0.0
2	MPRAGE	3T Tim Trio	2.4	3.16	8°	256 × 256 × 250	1	6.0.0
3	FSPGR	3T GE HDx	7.9	3.0	20°	256 × 256 × 176	1	6.0.0

FOV, field of view; FSPGR, 3-dimensional fast spoiled gradient echo sequence; MPRAGE, Magnetization Prepared-RApid Gradient Echo; TE, echo time; TR, repetition time.

### Morphometric score (MRS) analysis

**Fig. 1.**
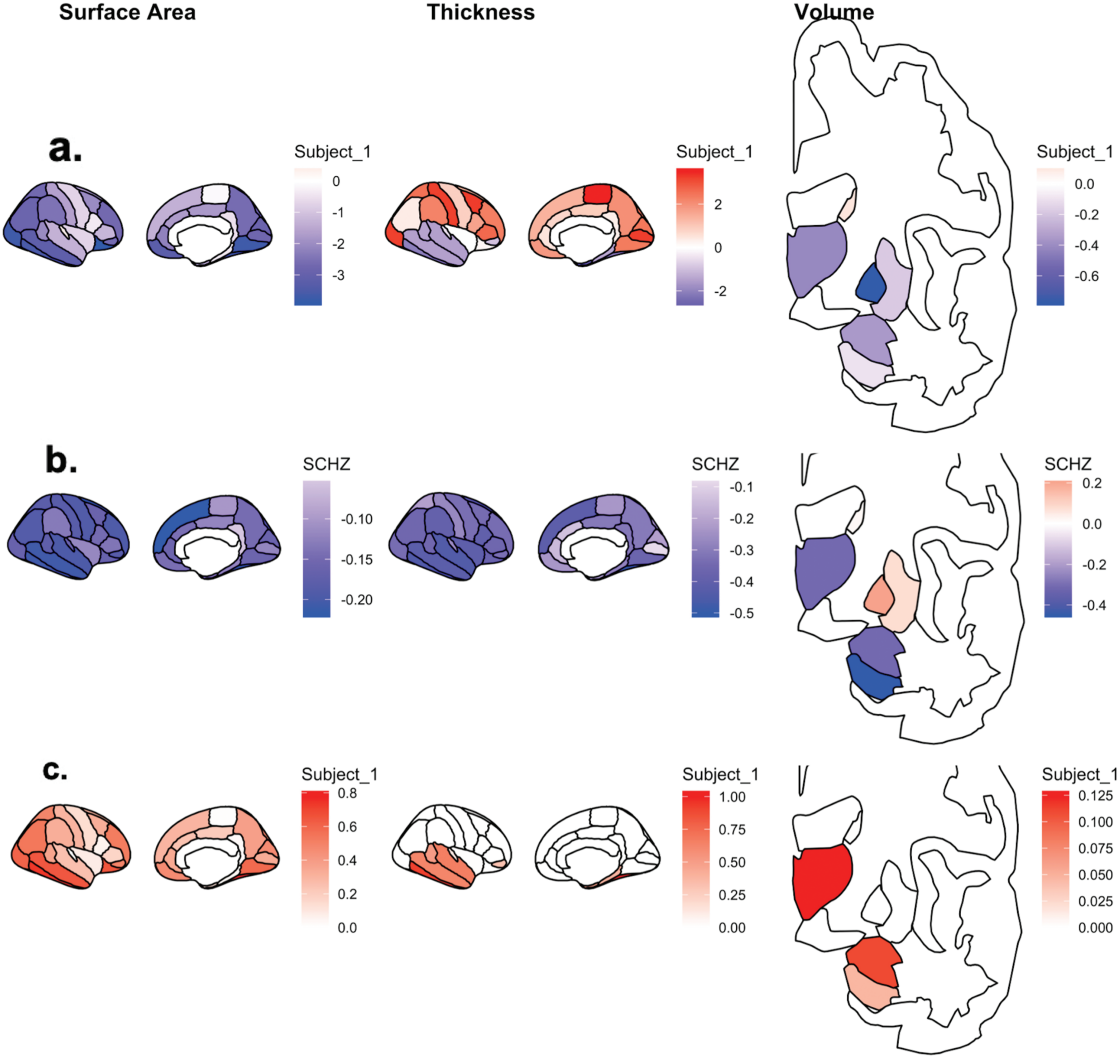
Morphometric score (MRS) analysis for an example participant. For each participant, (a) each of the 75 ROIs are covariate corrected and normalised into *z*-scores; (b) each *z*-transformed ROI is weighted by the corresponding ROI effect size (Cohen’s *d*) from meta-analysis provided by ENIGMA-SCZ working group; (c) in ROIs where the z-score and SCZ-ENIGMA effect sizes are congruently signed. The SCZ-MRS reflects an average across these weighted ROIs.


Measurements from 75 bilaterally averaged regions of interest (ROIS: 7 subcortical volume (mm^3^), 34 surface area (mm^2^); 34 thickness (mm)) were corrected for age, sex and intracranial volume (ICV) and normalised for each of the three samples, independently. Deconfounded ROIs were rescaled into standardised units to allow equal weighting amongst structural metric scales, enable outlier detection and permit future comparison across independent samples. For each subject, we considered the deviation of each ROI, compared to the distribution of the metric from the rest of the whole sample (across all SCZ patients/high PRS groups and controls) from which that subject was a participant. Each participant—ROI combination is then weighted by the effect size established from ENIGMA SCZ meta-analysis.^2,3^ Each ROI was weighted in the same direction (i.e. effect size increase/decrease) as observed in SCZ-cases versus controls in the SCZ-ENIGMA studies (supplementary Table 1). For example, the putamen is associated with a volumetric increase in SCZ cases compared to controls,^2^ so individuals with a larger putamen would have their putamen score weighted by the SCZ-ENIGMA respective effect size for putamen in SCZ cases (*d* = 0.37,^2^). If the ROI is smaller in SCZ cases, ROIs are weighted by the respective negative effect size. For each participant, we repeated this process for all 75 ROIs, accounting for each ROI sign. The absolute weighted ROIs were then averaged across all considered ROIs. As a negative control analysis, we also repeated this process, but omitted the weighting via ENIGMA effect sizes, to investigate the influence of the SCZ priors on the MRS. See Equation (1) for MRS formula and figure 1 for the schematic of MRS calculation from a *Z*-distribution of example subject.

$$\begin{array}{l} MRS_{i}=\underset{roi}{\overset{M}{\sum }}\;d_{ROI\;}\times \;\left(Z\;|\;sign(d)\;\right)\; \end{array}$$

**Equation 1.** For an individual (i), the morphometric risk score (MRS) represents the average of each of the ENIGMA-SCZ effect sizes (*d*_*ROI*_; N=75) multiplied by Z (each individual age, sex and ICV de-confounded region of interest) where Z and *d*_*ROI*_ are signed in a congruent manner.

### SCZ specific effects

To establish whether the SCZ-MRS profiles were specific to SCZ and not related to unspecific reductions in structural MRI metrics, we further examined individuals with attention hyperactivity deficient disorder (ADHD) who were recruited as part of the broader study detailed in Study 1 (*N* = 36; age: 32.81 ± 10.23); 18 female/18 male).

## Results

### Effect size comparisons

**Fig. 2.**
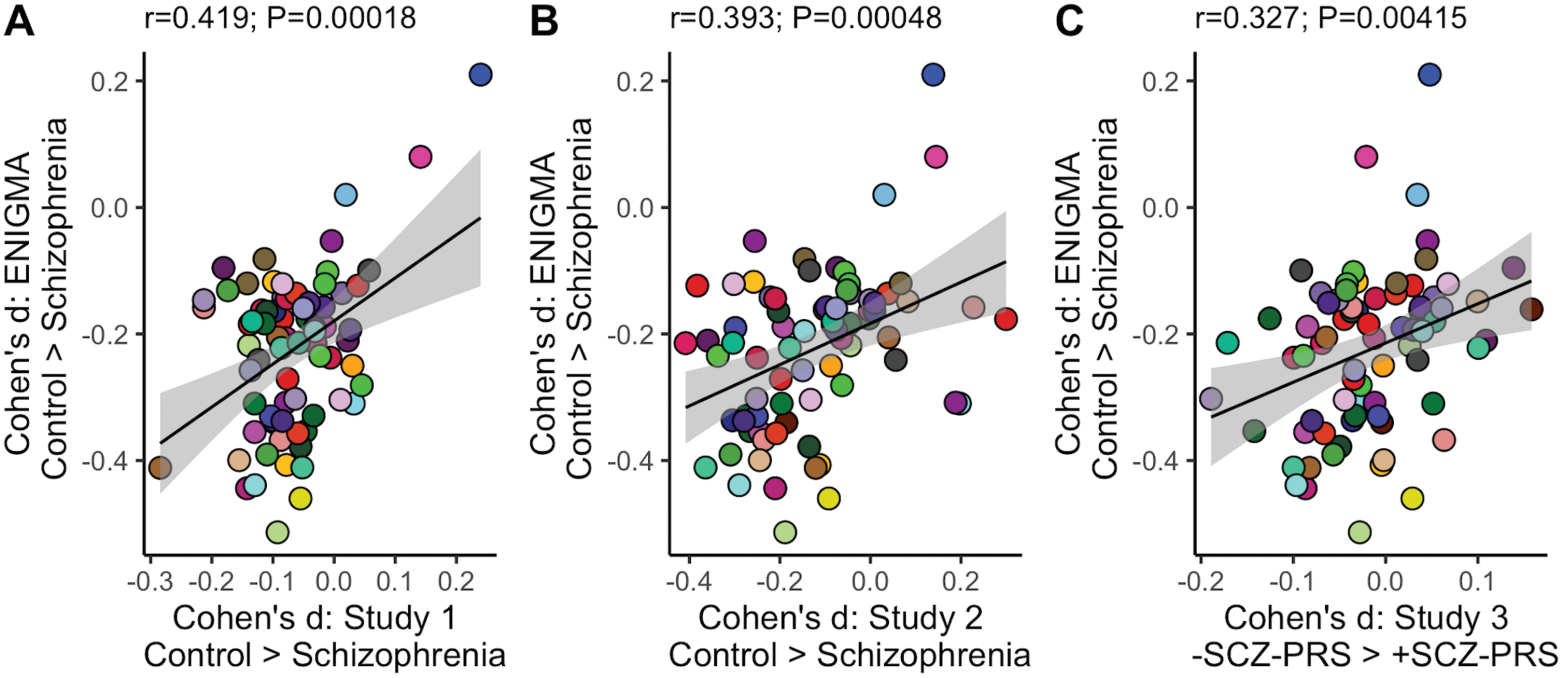
Each data point represents a SCZ adjusted effect size for a cortical (surface area and thickness)/subcortical (volume) region of interest (ROI; *N* = 75). Cohen’s *d =* standardised effect size. *Y*-axis = ENIGMA-SCZ; effect sizes derived from meta-analysis of healthy controls (HC) vs. schizophrenia cases (SCZ). A and B) HC vs. SCZ effect sizes derived from the independent SCZ case/HC groups C) RBG Low vs. High = effect sizes derived from comparison of healthy individuals based on SCZ-PRS.

Effect sizes for SCZ were consistent between ROIs observed in ENIGMA-SCZ and each of the three independent studies. These analyses ensured that the brain-wide impact of SCZ was comparable between ENIGMA-SCZ and our samples and supports the further weighting of SCZ-effect sizes in the MRS analysis. ROI effect sizes in the SCZ-ENIGMA were spatially correlated with those estimated in both the CNP Control vs. SCZ case analysis and CCNMD Control vs. SCZ case analysis and C) SCZ-PRS Low vs. High analysis (see figure 2). We observed one Bonferroni-corrected association between SCZ status and brain structure, adjusting for comparisons across all 75 ROIs (Study 1; middle temporal thickness: *d* = −0.28; *P*_BONFERRONI_ = 0.017), there were no Bonferroni-corrected associations between cortical thickness, surface area or volume and SCZ status in Study 2 or SCZ-PRS effects in Study 3, as per our prior study^26^). However, the brain-wide effects of SCZ on all ROIs were observed to an extent in all three samples at a whole brain level.

### MRS effects

**Fig. 3.**
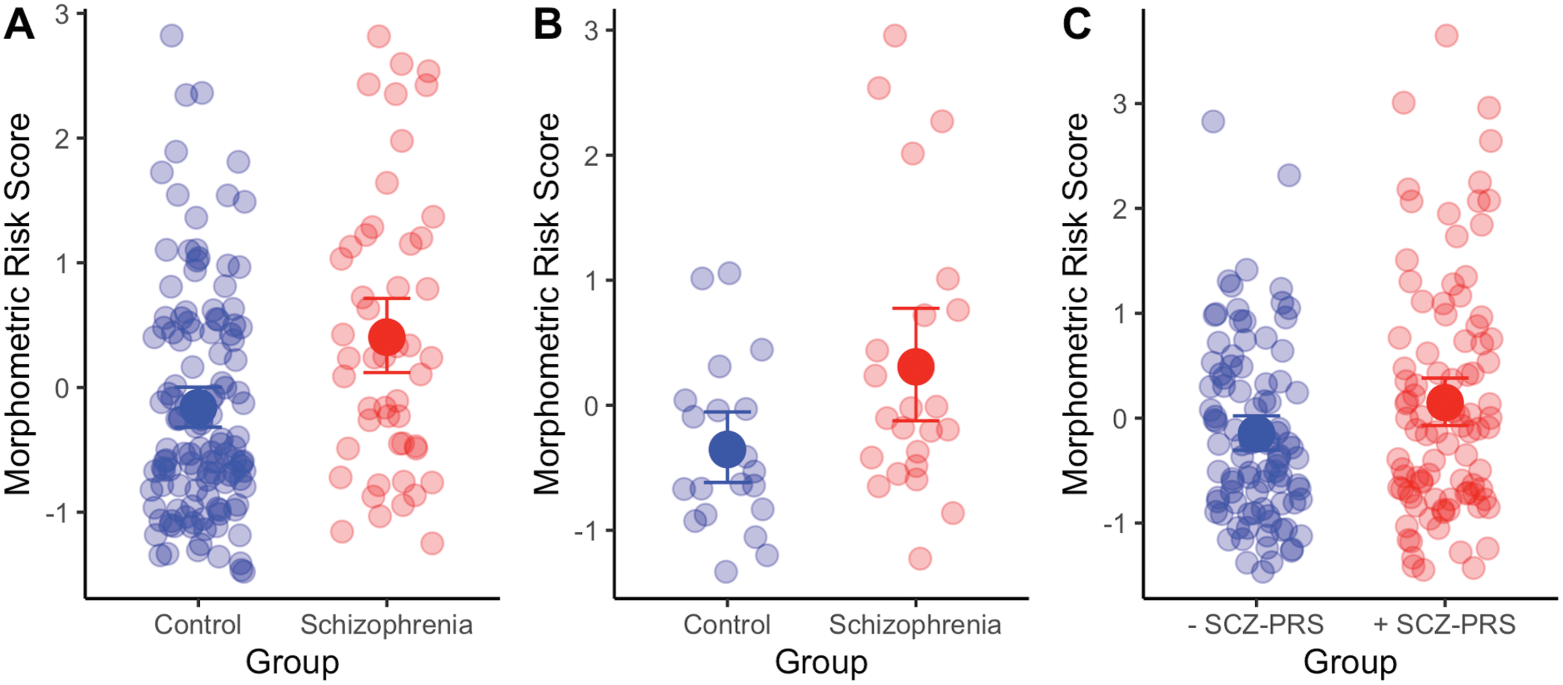
Group differences in morphometric score (MRS) for the (A) CNP [20]; (B) CMMND [21, 22] and (C) RBG [26] data sets. Error bars represent 95% bootstrapped confidence intervals.

The SCZ group had higher MRS than the control samples in both of case/control studies (Study 1: β = 0.621 [95% CIs = 0.284–0.958]; *P* = 0.0004; Study 2: β = 0.806 [95% CIs = 0.169–1.443]; *P* = 0.0179 (figure 3). For Study 1, we also had additional SCZ spectrum/subtype information (Undifferentiated; Paranoid; Residual & Schizoaffective: *N* = 11; 21; 6; 11, respectively). The largest SCZ-MRS group difference was between controls and the Schizoaffective subgroup (*P*_BONFERRONI_ = 0.012). In Study 3, the high SCZ-PRS group also had a higher MRS than the low SCZ-PRS group (*β* = 0.294 [95% CIs = 0.012–0.576]; *P* = 0.044). A control analysis that omitted the SCZ ROI effect size weighting from the MRS analysis failed to delineate a group difference across all three studies (Study 1: *β* = 0.27, *P* = 0.12; Study 2: *β* = 0.31, *P* = 0.33; Study 3: *β* = 0.24, *P* = 0.08) suggesting that SCZ weights shaped the estimation of a SCZ relevant MRS profile.

### Cross disorder analysis

**Fig. 4.**
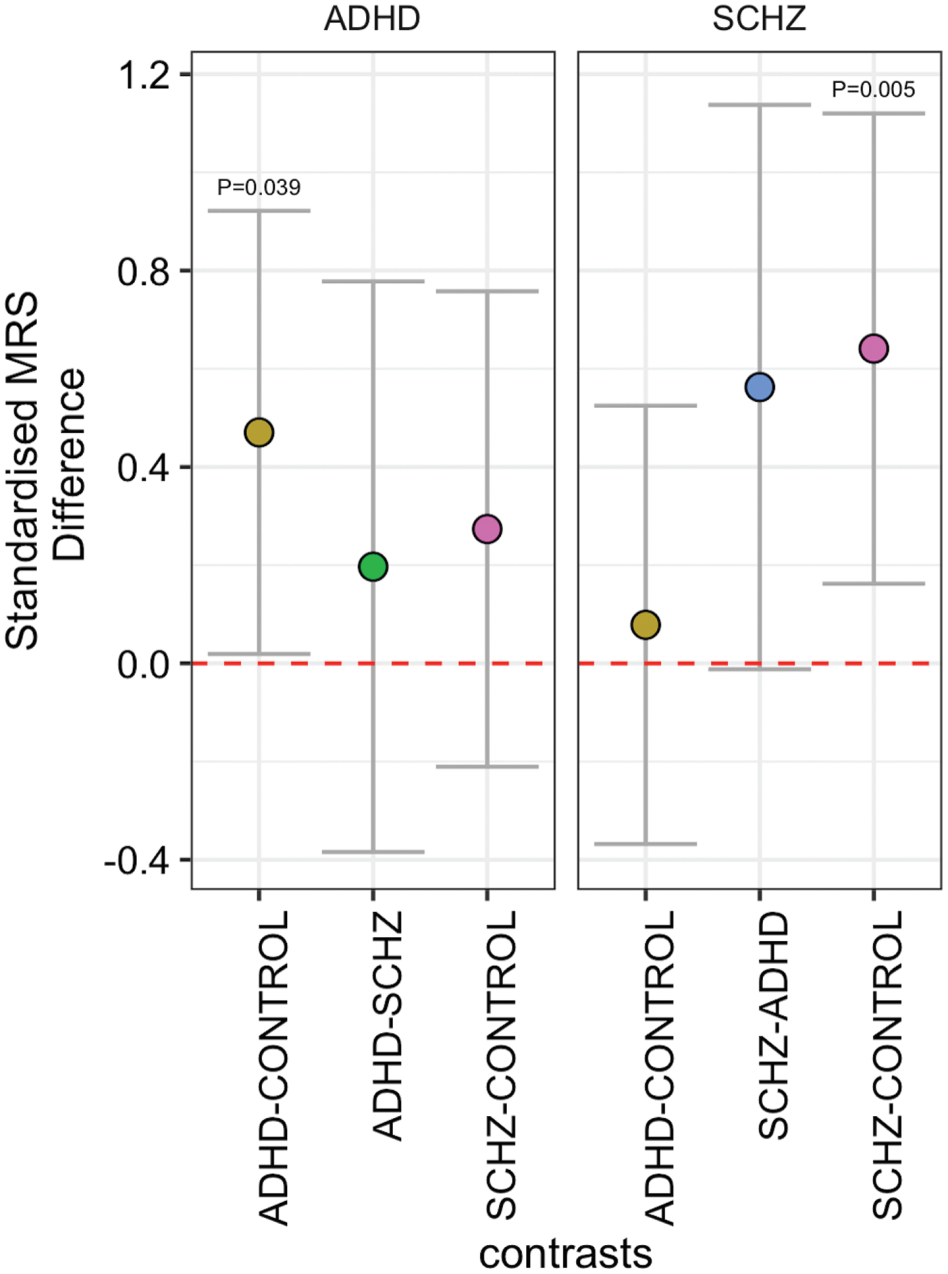
Standardised effect sizes for comparisons across diagnostic groups for left) ADHD and right) SCZ MRS, across the extended sample. *P* values highlighted are adjusted via Bonferroni correction. Error bars represent 95% confidence intervals of the beta estimate.

We repeated the MRS analysis across the expanded cohort, with the additional use of ADHD weights (Cohen’s *d* effect sizes) from recent meta-analysis studies of ADHD on subcortical volume, cortical thickness and surface area.^32,33^ In the expanded sample (*N*_ADHD_ = 36; *N*_HEATHY CONTROLS_ = 110; *N*_SCZ_ = 50), SCZ-MRS was specifically associated with SCZ case status (*β* = 0.61; *P*_BONFERRONI_ = 0.005) while the ADHD-MRS was related to ADHD case status (*β* = 0.48; *P*_BONFERRONI_ = 0.039). No other case–case or case–control comparison survived correction for multiple comparisons (figure 4).

### Cognition and symptom analysis

We combined data on cognitive ability for all participants across study 1–2 (*N*_COMBINED_ = 220) using data from the Wechsler Adult Intelligence Scale (WAIS-IV) for indices of working memory, verbal comprehension and perceptual reasoning.^34^ We observed a negative association between SCZ-MRS and working memory (Letter Number Sequencing), adjusted for the covariates and corrected across all metrics (*β* = −0.068, *P*_BONFERRONI_ = 0.032). Global factors in the Scale for the Assessment of Positive and Negative Symptoms (SAPS, SANS)^35,36^ were also available for SCZ cases across study 1–2 (*N*_COMBINED_ = 73). We did not observe any associations between SANS/SAPS factors and SCZ-MRS that corrected for multiple comparisons. However, most effects were in the hypothesised direction (SCZ-MRS associated with lower cognition and higher symptom scores: P_SIGN.TEST_=0.038). See Supplementary Figure 1/Supplementary Table 2 for further information.

## Discussion

Schizophrenia (SCZ) is associated with volumetric, surface area and thickness differences across the brain, with varying effect sizes of SCZ status on individual cortical or subcortical regions. SCZ at-risk groups such as relatives of SCZ and SCZ-PRS carriers also demonstrate small effects across discrete metrics of brain structure.^37–41^ However, little work has assessed the relationship between SCZ-PRS and an individual’s cumulative SCZ-related structural brain alterations above a conventional univariate approach.

Here, we demonstrate the efficacy of a novel method to address inter and intra-individual variation in brain structure, by generating a risk score reflecting individual proclivity for SCZ-related brain changes. For each participant, we weighted each of the 75 ROIs by the extent to which they deviated from a normative model by the effect sizes provided by ENIGMA-SCZ working group effect size estimate to compose an SCZ-MRS score. To our knowledge, this is the first approach to use prior metrics (e.g. ROI ENIGMA SCZ effect sizes) to cumulatively weight novel discovery data. Prior normative modelling approaches have used non-weighted techniques, which have also been linked to SCZ and genetic liability SCZ-PRS,^6^ but these approaches do not consider prior effect sizes such as SCZ-ENIGMA to weight ROIs that deviated from the normative sample, and thus would not permit more weighting to cortical regions with a more prominent alterations in SCZ and may not estimate the disorder-specific effects we observed in the present study. We also note that similar metrics such as the regional-vulnerability index (RVI) also demonstrate SCZ/control differences, cognitive correlates and disorder specificity.^14–17^ Our MRS differs from this approach as we assess each ROI independently, rather than correlating all ROI/meta-analysis effect sizes, per individual. Future studies empirically comparing the sensitivity and specificity of these techniques are warranted. We also observed that the SCZ-MRS was further linked to a schizoaffective phenotype and reductions in working memory. Recent multivariate studies exploring cognitive correlates of schizophrenia-related structural brain features have also implicated cognitive dimensions such as working memory,^16,42,43^ suggesting alterations manifest across wider brain-wide networks.

While cognitive deficits have linked to structural alterations across a wider SCZ spectrum,^42,44,45^ future work will be required to validate differences in SCZ-MRS profiles across SCZ subtypes.

Our findings should be interpreted with the following limitations. First, we did not have genomic data for the CNP and CCMND cohorts. This study would have benefitted from this data as it would have allowed us to make inferences regarding shared or interactive relationships between SCZ-PRS and SCZ-MRS on SCZ diagnostic outcomes. Further studies that incorporate cohorts with SCZ case/healthy controls, genetics and MRI data will be helpful to understand the impact of MRS and the combined/ interactive effects of PRS and MRS. Second, analogous to sources of bias in PRS such as population stratification and transethnic performance,^46^ the MRS may be susceptible to bias, based on the sample/instruments used as training data. This could potentially lead to inflation of MRS (i.e. MRS may under-perform when samples/instruments are different). This that SCZ-MRS are likely to be more predictive of a SCZ phenotype that was collected in the meta-analysis from which our weights were derived.^2,3^ Third, to generate our MRS, we assign SCZ-ENIGMA weights based on a normative atlas of ROIs. More sophisticated solutions such as voxel/vertex-wise approaches may also help to capture inter-subject SCZ-like morphometric profiles. Future studies to refine the MRS will require multi-site collaboration and leave-one-out strategies to understand optimal approaches for profiling individual subject SCZ brain profiles. Fourth, our SCZ-MRS metric shows a considerable overlap between SCZ patients/ high SCZ-PRS groups and control samples, making it a suitable assessment for discrete groups/ independent samples, rather than individuals or groups consisting of only SCZ patients. However, as population-wide normative MRI data becomes more available,^47^ our SCZ-MRS may offer clinical/diagnostic utility beyond the context of the sample from which they are acquired. Fifth, we acknowledge that our low SCZ-PRS group may not reflect the MRS of individuals from the general population as they reflect a sample of individuals with an extremely low PRS for SCZ, rather than a population average. Finally, we note that the correlation between ENIGMA SCZ effect sizes and effect sizes from our three studies were smaller than prior effect size correlations.^16^ We would suggest that this could be due to factors such as (i) sample size and (ii) inclusion of surface area in our MRS which has a less pronounced SCZ-related phenotype,^3^ compared to multi-modal imaging approaches that incorporate white matter microstructure alterations.^16,48^

In conclusion, we employ a multivariate approach for assessing brain-wide alterations in structural MRI samples to show that both SCZ cases and healthy individuals with high SCZ-PRS show increased proclivity for SCZ-related brain changes, using effect sizes from prior, independent meta-analysis. Our observations are supported by evidence that SCZ effects on MRI metrics are reproducible and consistent in smaller, independent samples across a brain-wide topology. This was established as SCZ-ENIGMA ROI-wise effect sizes were correlated with ROI effect sizes in all three samples, indicating that SCZ–related brain changes were present globally, across the whole brain, in both SCZ cases and high SCZ-PRS groups. These consistent observations demonstrate that, while small to moderate-sized samples may not be powered to detect SCZ-related brain changes using conventional univariate approaches, the broad range of SCZ effect sizes found in such smaller samples is comparable to ENIGMA-SCZ meta-analysis, and that SCZ-related effects are consistent across a wider brain topology. This also supports our MRS approach by demonstrating that ENIGMA-SCZ effects provide informative priors over and above the null hypothesis. Our approach has potential utility for cross-modal MRI applications, for any psychiatric condition with well-established brain changes^1^ and helps to parse patient heterogeneity not reflected in large meta-analytical SCZ case/control neuroimaging studies.^6,38,49–51^ We expect it can also aid future efforts to improve diagnostic classification or prediction based on a combination of biological (e.g. MRS in addition to PRS), psychometric and clinical metrics.

## Supplementary Material

sbab125_suppl_Supplementary_Materials
