# Supplementary material for: Morphometric Analysis of Structural MRI Using Schizophrenia Meta-analytic Priors Distinguish Patients from Controls in Two Independent Samples and in a Sample of Individuals With High Polygenic Risk
Source: Schizophr Bull. 2021 Oct 18;48(2):524–32. doi: 10.1093/schbul/sbab125 (PMC8886591; doi:10.1093/schbul/sbab125)
Supplement: sbab125_suppl_Supplementary_Materials [file sbab125_suppl_supplementary_materials.docx]

**Study 3: ALPSAC cohort and genotyping**

Individuals recruited via the wider ALSPAC sample ^1, 2^. Briefly, Pregnant women resident in Avon, UK with expected dates of delivery 1st April 1991 to 31st December 1992 were invited to take part in the study. The initial number of pregnancies enrolled is 14,541 (for these at least one questionnaire has been returned or a “Children in Focus” clinic had been attended by 19/07/99). Of these initial pregnancies, there was a total of 14,676 foetuses, resulting in 14,062 live births and 13,988 children who were alive at 1 year of age. Participants were genotyped using the Illumina HumanHap550 quad chip genotyping platforms by 23andme subcontracting the Wellcome Trust Sanger Institute, Cambridge, UK and the Laboratory Corporation of America, Burlington, NC. Ethical approval for the study was obtained from the ALSPAC Ethics and Law Committee and the Local Research Ethics Committees. Consent for biological samples has been collected in accordance with the Human Tissue Act (2004). The genome-wide data were subjected to quality control as follows: individuals were excluded on the basis of sex mismatches; minimal or excessive heterozygosity; individual SNP missingness (>3%). Population stratification was assessed by multidimensional scaling analysis and compared with Hapmap II (release 22) European descent (CEU), Han Chinese, Japanese, and Yoruba reference populations; all individuals with non-European ancestry were removed. SNPs with a minor allele frequency of <1%, a call rate of <95% or evidence for violations of Hardy-Weinberg equilibrium (*P* < 5E-7) were removed. Cryptic relatedness was measured/excluded as proportion of identity by descent (IBD > 0.1). Related subjects that passed all other quality control thresholds were retained during subsequent phasing and imputation. Nine thousand one hundred fifteen subjects and 500527 SNPs passed these quality control filters. We combined 477482 SNP genotypes in common between the sample of mothers and sample of children. We removed SNPs with genotype missingness above 1% due to poor quality (11396 SNPs removed) and removed a further 321 subjects due to potential ID mismatches, resulting in a data set containing 465740 SNPs. We estimated haplotypes using ShapeIT (v2.r644) which utilizes relatedness during phasing. We obtained a phased version of the 1000 genomes reference panel (phase 1, version 3) from the Impute2 reference data repository (phased using ShapeIt v2.r644, haplotype release date Dec 2013). Imputation of the target data was performed using Impute V2.2.2 against the reference panel (all polymorphic SNPs excluding singletons), using all 2186 reference haplotypes (including non-Europeans). After quality control, a total of 8365 individuals were genotyped and underwent SCZ-PRS calculations. Please note that the study website contains details of all the data that is available through a fully searchable data dictionary and variable search tool available at <http://www.bristol.ac.uk/alspac/researchers/our-data/>.

**Supplementary Table 1.** Effect sizes (Cohen’s *d*), averaged across hemisphere for cortical thickness (_thickavg); cortical surface area (_surfavg) and volume for Schizophrenia (SCHZ, from ^3, 4^) and Attention Deficits Hyperactivity Disorder (ADHD, from ^5, 6^).

| Region_of_interest | ADHD | SCHZ |
| --- | --- | --- |
| Accumbensarea | -0.15 | -0.25 |
| Amygdala | -0.19 | -0.31 |
| Caudate | -0.11 | 0.02 |
| Hippocampus | -0.11 | -0.46 |
| Pallidum | 0.00 | 0.21 |
| Putamen | -0.14 | 0.08 |
| ThalamusProper | -0.03 | -0.31 |
| bankssts_thickavg | 0.00 | -0.3535 |
| caudalanteriorcingulate_thickavg | -0.04 | -0.1355 |
| caudalmiddlefrontal_thickavg | -0.05 | -0.34 |
| cuneus_thickavg | 0.02 | -0.2145 |
| entorhinal_thickavg | -0.08 | -0.1765 |
| frontalpole_thickavg | 0.01 | -0.2105 |
| fusiform_thickavg | -0.10 | -0.5135 |
| inferiorparietal_thickavg | 0.01 | -0.355 |
| inferiortemporal_thickavg | -0.03 | -0.444 |
| insula_thickavg | -0.05 | -0.407 |
| isthmuscingulate_thickavg | 0.03 | -0.3085 |
| lateraloccipital_thickavg | 0.03 | -0.3365 |
| lateralorbitofrontal_thickavg | -0.03 | -0.378 |
| lingual_thickavg | -0.02 | -0.3675 |
| medialorbitofrontal_thickavg | 0.00 | -0.2375 |
| middletemporal_thickavg | -0.02 | -0.4115 |
| paracentral_thickavg | -0.05 | -0.2345 |
| parahippocampal_thickavg | -0.06 | -0.281 |
| parsopercularis_thickavg | -0.04 | -0.3995 |
| parsorbitalis_thickavg | -0.02 | -0.329 |
| parstriangularis_thickavg | 0.01 | -0.357 |
| pericalcarine_thickavg | -0.01 | -0.0815 |
| postcentral_thickavg | -0.02 | -0.271 |
| posteriorcingulate_thickavg | -0.03 | -0.304 |
| precentral_thickavg | -0.11 | -0.33 |
| precuneus_thickavg | -0.03 | -0.3025 |
| rostralanteriorcingulate_thickavg | -0.01 | -0.151 |
| rostralmiddlefrontal_thickavg | 0.00 | -0.3375 |
| superiorfrontal_thickavg | 0.00 | -0.411 |
| superiorparietal_thickavg | 0.01 | -0.214 |
| superiortemporal_thickavg | 0.00 | -0.439 |
| supramarginal_thickavg | -0.02 | -0.3905 |
| temporalpole_thickavg | -0.12 | -0.241 |
| transversetemporal_thickavg | 0.01 | -0.2575 |
| bankssts_surfavg | -0.07 | -0.1755 |
| caudalanteriorcingulate_surfavg | -0.09 | -0.142 |
| caudalmiddlefrontal_surfavg | -0.13 | -0.161 |
| cuneus_surfavg | -0.06 | -0.1625 |
| entorhinal_surfavg | -0.07 | -0.1235 |
| frontalpole_surfavg | -0.05 | -0.0955 |
| fusiform_surfavg | -0.12 | -0.218 |
| inferiorparietal_surfavg | -0.11 | -0.1885 |
| inferiortemporal_surfavg | -0.10 | -0.2065 |
| insula_surfavg | -0.12 | -0.1175 |
| isthmuscingulate_surfavg | -0.09 | -0.053 |
| lateraloccipital_surfavg | -0.11 | -0.164 |
| lateralorbitofrontal_surfavg | -0.12 | -0.1645 |
| lingual_surfavg | -0.07 | -0.158 |
| medialorbitofrontal_surfavg | -0.11 | -0.144 |
| middletemporal_surfavg | -0.10 | -0.2055 |
| paracentral_surfavg | -0.04 | -0.102 |
| parahippocampal_surfavg | -0.08 | -0.121 |
| parsopercularis_surfavg | -0.08 | -0.1485 |
| parsorbitalis_surfavg | -0.08 | -0.1835 |
| parstriangularis_surfavg | -0.12 | -0.136 |
| pericalcarine_surfavg | -0.08 | -0.12 |
| postcentral_surfavg | -0.10 | -0.1845 |
| posteriorcingulate_surfavg | -0.13 | -0.121 |
| precentral_surfavg | -0.08 | -0.1905 |
| precuneus_surfavg | -0.10 | -0.1465 |
| rostralanteriorcingulate_surfavg | -0.11 | -0.1575 |
| rostralmiddlefrontal_surfavg | -0.12 | -0.1935 |
| superiorfrontal_surfavg | -0.15 | -0.222 |
| superiorparietal_surfavg | -0.13 | -0.18 |
| superiortemporal_surfavg | -0.11 | -0.1955 |
| supramarginal_surfavg | -0.09 | -0.131 |
| temporalpole_surfavg | -0.11 | -0.0995 |
| transversetemporal_surfavg | -0.06 | -0.1595 |

**Supplementary Figure 1 / Table 2.** Association between SCZ-MRS and Wechsler Adult Intelligence Scale (WAIS-IV) subsets and global factors for the Scale for the Assessment of Positive and Negative Symptoms (SANS, SAPS). All estimates are adjusted for covariates within sample. Error bars represent 95% confidence intervals of the beta estimate. Combined effects were estimated via fixed-effect meta-analysis.


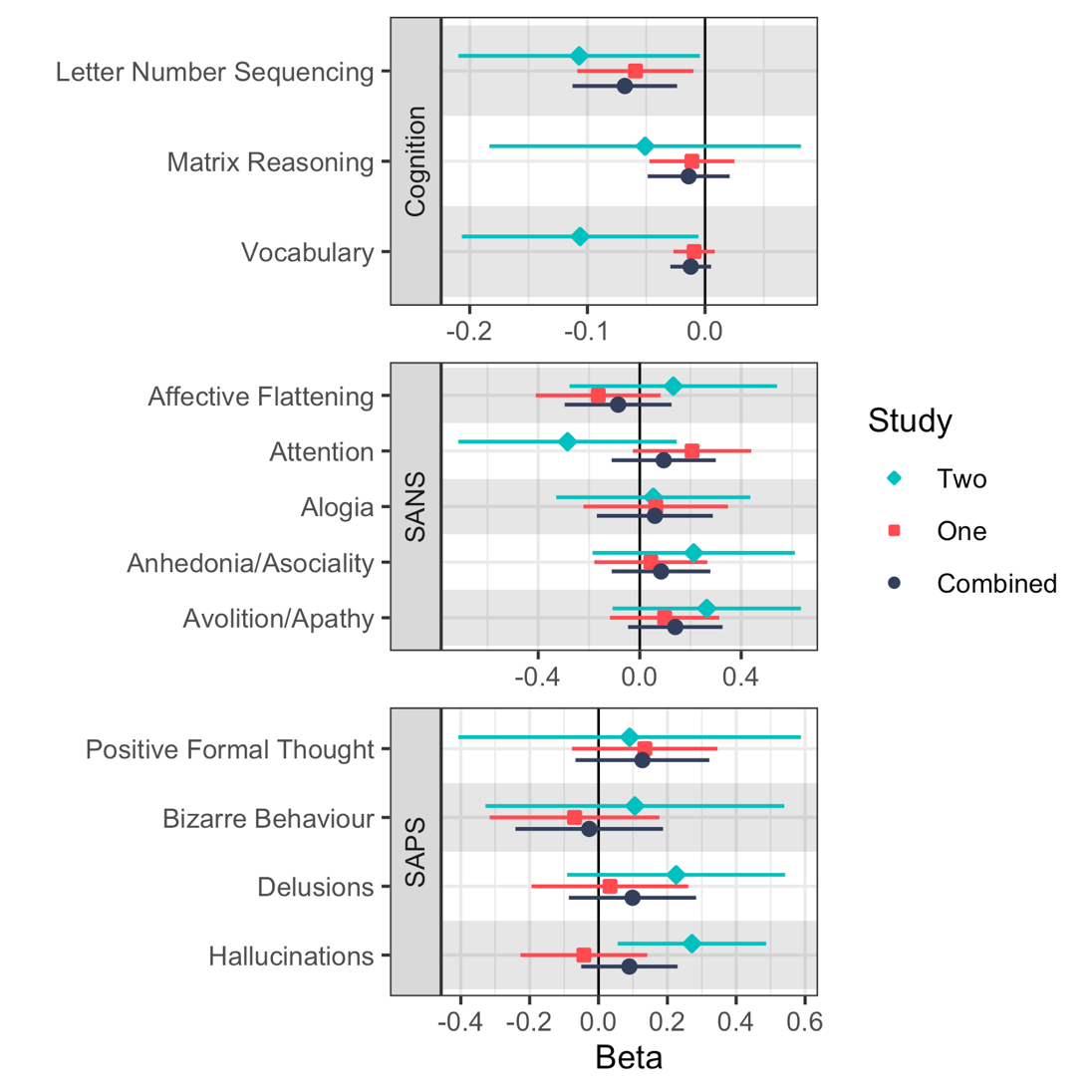


| COMBINED | | | | | |
| --- | --- | --- | --- | --- | --- |
| TRAIT | PHENOTYPE | BETA | SE | PVAL | P_BON_ |
| Cognition | Letter Number Sequencing | -0.068 | 0.023 | 0.003 | 0.032 |
| Cognition | Matrix Reasoning | -0.014 | 0.018 | 0.434 | 1.000 |
| Cognition | Vocabulary | -0.012 | 0.009 | 0.169 | 1.000 |
| SANS | Affective Flattening | -0.085 | 0.108 | 0.429 | 1.000 |
| SANS | Attention | 0.094 | 0.105 | 0.367 | 1.000 |
| SANS | Alogia | 0.059 | 0.117 | 0.610 | 1.000 |
| SANS | Anhedonia/Asociality | 0.084 | 0.099 | 0.398 | 1.000 |
| SANS | Avolition/Apathy | 0.140 | 0.095 | 0.141 | 1.000 |
| SAPS | Positive Formal Thought | 0.128 | 0.099 | 0.199 | 1.000 |
| SAPS | Bizarre Behaviour | -0.027 | 0.109 | 0.806 | 1.000 |
| SAPS | Delusions | 0.099 | 0.094 | 0.295 | 1.000 |
| SAPS | Hallucinations | 0.090 | 0.072 | 0.210 | 1.000 |

**Supplementary References**

**1.** Boyd A, Golding J, Macleod J, et al. Cohort Profile: the 'children of the 90s'--the index offspring of the Avon Longitudinal Study of Parents and Children. *International journal of epidemiology* Feb 2013;42(1):111-127.

**2.** Fraser A, Macdonald-Wallis C, Tilling K, et al. Cohort Profile: the Avon Longitudinal Study of Parents and Children: ALSPAC mothers cohort. *International journal of epidemiology* Feb 2013;42(1):97-110.

**3.** van Erp TG, Hibar DP, Rasmussen JM, et al. Subcortical brain volume abnormalities in 2028 individuals with schizophrenia and 2540 healthy controls via the ENIGMA consortium. *Mol Psychiatry* Apr 2016;21(4):547-553.

**4.** van Erp TGM, Walton E, Hibar DP, et al. Cortical Brain Abnormalities in 4474 Individuals With Schizophrenia and 5098 Control Subjects via the Enhancing Neuro Imaging Genetics Through Meta Analysis (ENIGMA) Consortium. *Biological psychiatry* Nov 1 2018;84(9):644-654.

**5.** Hoogman M, Bralten J, Hibar DP, et al. Subcortical brain volume differences in participants with attention deficit hyperactivity disorder in children and adults: a cross-sectional mega-analysis. *Lancet Psychiatry* Apr 2017;4(4):310-319.

**6.** Hoogman M, Muetzel R, Guimaraes JP, et al. Brain Imaging of the Cortex in ADHD: A Coordinated Analysis of Large-Scale Clinical and Population-Based Samples. *Am J Psychiatry* Jul 1 2019;176(7):531-542.
